# End-point kinematics using virtual reality explaining upper limb impairment and activity capacity in stroke

**DOI:** 10.1186/s12984-019-0551-7

**Published:** 2019-07-01

**Authors:** Netha Hussain, Katharina S. Sunnerhagen, Margit Alt Murphy

**Affiliations:** Institute of Neuroscience and Physiology, Sahlgrenska Academy, University of Gothenburg, Per Dubbsgatan 14, 3rd Floor, SE-41345 Gothenburg, Sweden

**Keywords:** Stroke rehabilitation, Upper extremity movement, Kinematics, Outcome assessment, Virtual reality

## Abstract

**Background:**

For evaluation of upper limb impairment and activity capacity, Fugl-Meyer Assessment of Upper Extremity (FMA-UE) and Action Research Arm Test (ARAT) are recommended to be included in stroke trials. To improve the understanding of mechanisms of motor recovery, and differentiate between restitution and compensation, kinematic analysis is also recommended for assessment of upper limb function after stroke.

**Aim:**

To determine the extent to which end-point kinematic variables obtained from the target-to-target pointing task were associated with upper limb impairment or activity limitation as assessed by traditional clinical scales in individuals with stroke.

**Methods:**

Sixty-four individuals, from acute stage up to one year after stroke, were included from the Stroke Arm Longitudinal study at the University of Gothenburg (SALGOT) cohort. They performed a target-to-target pointing task in a virtual environment using a haptic stylus which also captured the kinematic parameters. Multiple linear regression was done to determine the amount of variance explained by kinematic variables on FMA-UE and ARAT scores after controlling for confounding variables.

**Results:**

Mean velocity and number of velocity peaks explained 11 and 9% of the FMA-UE score uniquely and 16% when taken together. Movement time and number of velocity peaks explained 13 and 10% of the ARAT score respectively.

**Conclusion:**

The kinematic variables of movement time, velocity and smoothness explain only a part of the variance captured by using clinical observational scales, reinforcing the importance of multi-level assessment using both kinematic analysis and clinical scales in upper limb evaluation after stroke.

**Trial registration:**

The trial was registered with register number NCT01115348 at clinicaltrials.gov, on May 4, 2010. URL: https://clinicaltrials.gov/ct2/show/NCT01115348.

## Background

In stroke, the upper limb function is impaired in approximately 50–80% of individuals in the acute phase [1–3], and 40–50% in the chronic phase [2, 4]. Stroke being the leading cause of disability in adults [5] and upper limb impairment being one of the most relevant functions affected in stroke [6], rehabilitation of the upper limb in individuals after stroke gathers prime importance. The goal of upper limb rehabilitation is to improve the functional use of the arm so that the individual becomes capable to perform productive activities in real life. In order to achieve this goal, it is important to assess the arm function and activity capacity on a periodic basis in individuals after stroke, so that the course of rehabilitation interventions can be planned accordingly. Body functions (negative term impairment) and activity (negative term activity limitations) are two of the three levels by which health conditions are classified in the International Classification of Functioning, Disability and Health (ICF) [7]. The activity is further divided into activity capacity, which indicates what a person can do e.g. in a test situation, and into activity performance, which indicates what a person is doing as daily routine.

The recommended clinical scales for assessment of upper limb sensorimotor impairment and activity capacity limitation in stroke trials are Fugl-Meyer Assessment of Upper Extremity (FMA-UE) and Action Research Arm Test (ARAT) respectively [8]. Recently, there has been a shift towards multilevel assessment, which has stressed the need for inclusion of kinematics in stroke trials in order to understand the extent of upper limb impairment, the mechanisms of motor recovery and to allow differentiation between restitution and compensation [8]. Understanding the associations between clinical and kinematic measures would enable the identification of kinematic variables that best reflect sensorimotor impairment and activity limitation, thereby leading to better clinical interpretation of kinematic data.

Past studies have shown that kinematic variables from arm-supported robotic devices [9–12] and camera-based systems [13–18] partially explain the scores of clinical scales. As an extension to this line of research, the present study examines the association between kinematic variables obtained from a target-to-target pointing task using a haptic device and upper extremity impairment and activity capacity limitation in individuals with stroke. In this study, the haptic device allows for capturing kinematic variables from free arm movements in 3D. Although haptic devices have been increasingly used in rehabilitation settings, past studies using haptic devices had either small sample sizes [19, 20], dealt with conditions other than stroke [20–22] or examined tasks other than pointing [23]. So, the knowledge is limited about how kinematics obtained from the haptic device allowing free movements in a 3D space reflect upper limb functioning as assessed with clinical scales in individuals with stroke.

The target-to-target pointing task as used in this study is ecologically valid since it reflects a skill that is commonly used in a variety of day-to-day activities such as using a touch screen, pushing a key on a keyboard and pressing a switch. Thus, improved knowledge regarding the relationship between kinematic measures and clinical scales may provide further insights for the selection of outcome measures for evaluating the upper limb in individuals after stroke.

The aim of this study was to determine the extent to which end-point kinematic variables obtained from the target-to-target pointing task were associated with upper limb impairment or activity limitation as assessed by traditional clinical scales in individuals with stroke. Hypothetically, kinematic measures, such as movement time, smoothness and velocity can be considered to measure aspects of impairment according to ICF and therefore be expected to have stronger association with upper limb impairment than activity capacity assessments.

## Methods

### Subjects and design

Data for this study was extracted from Stroke Arm Longitudinal Study at Gothenburg University (SALGOT) [24], a longitudinal, prospective, observational study. The inclusion criteria were as follows: age over 18 years, living in Gothenburg urban area, first-ever clinical stroke, presence of stroke-related upper limb impairment, admitted to the stroke unit at Sahlgrenska University Hospital within three days of onset of stroke [24]. The exclusion criteria were: any upper limb condition prior to stroke that limits the functional use of the arm, severe multi-impairment or diminished physical condition before the stroke that will affect arm function, life expectancy less than 12 months due to other illnesses, not Swedish speaking prior to the stroke incident. In the SALGOT-study, a battery of assessment tests was done on each individual at 3 days, 10 days, 3,4 and 6 weeks and 3,6 and 12 months after the onset of stroke [24].

**Fig. 1:**
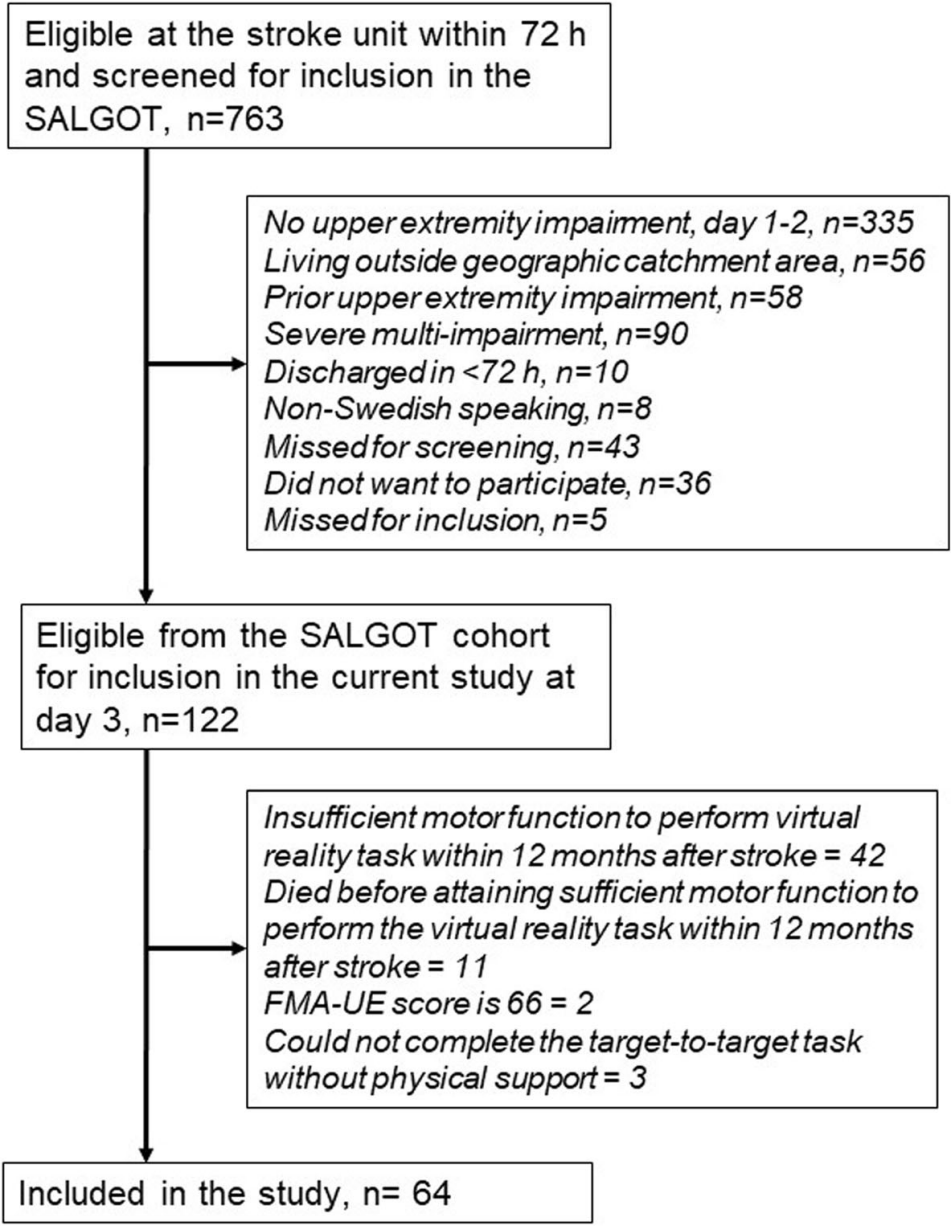
Flowchart of the inclusion process

All participants from SALGOT cohort who had sufficient motor function to perform the target-to-target task were included in the study. The lowest recorded FMA-UE score among the included participants was 32, implying that only those with moderate or mild stroke impairment had sufficient motor function to perform the target-to-target task. In addition, the participant should not have reached a full score of 66 points on the FMA-UE scale at the time of testing. Since the pointing task was performed at more than one time-point in most cases, the kinematic data from the first occasion only were included in the study. Figure 1 shows the flowchart of the inclusion process.

The SALGOT study protocol that included the present study was approved by the Regional Ethical Review Board in Gothenburg, Sweden (No. 225–08). All participants gave informed, written consent prior to their participation in the study.

### Equipment

**Fig. 2:**
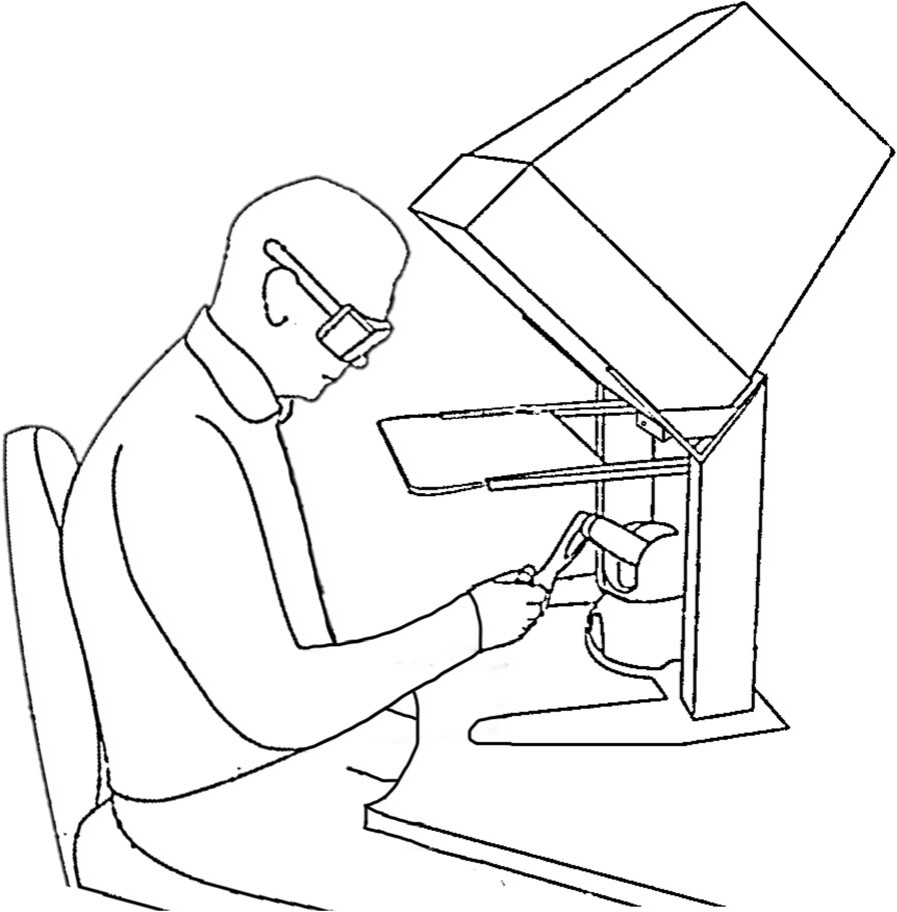
A participant performing the target-to-target pointing task

The virtual reality (VR) environment is a semi-immersive workbench in which 3D display of virtual space can be seen by the user on a mirror after wearing stereographic shuttered glasses (Fig. 2). An infrared transmitter on the workbench synchronized the image sequence, giving the participant an illusion of seeing 3D objects.

Kinematic data were captured by PHANTOM® Omni™ haptic device [25]. It included a stylus that has 6 degrees of freedom. The haptic stylus could be moved freely in the virtual workspace (220 × 140 × 120 mm). The device was auto-calibrated when the stylus was placed in its resting position. The user received force feedback and the target changed its color when the stylus was brought close the target. This setup, in addition to visual feedback created an illusion of touching the virtual target.

### Set-up and procedure

The kinematic data were gathered from the haptic device during the target-to-target pointing task. Sitting in front of the computer screen and wearing the 3D glasses, the participant held the stylus with one hand to reach and point at a green colored, disc shaped target (~ 3.0° viewing angle) in the virtual space. Each target disappeared when pointed at and a new target appeared in a new location. The participants needed to reach each target with the tip of the stylus in order to complete the task. The task, which consisted of 32 targets, ended when the last of all targets disappeared. The participant was asked to finish the task as fast and as accurately as possible.

### Kinematic variables

The kinematic variables were calculated using Curictus™ and MATLAB^®^ software. The data, sampled at 50 Hz, were filtered with a 6-Hz low pass second order Butterworth filter in both forward and backward directions. The entity between two adjacent targets was defined as a movement segment. A movement segment was defined as time when a target appears to time when it disappears. Five kinematic variables were calculated: movement time, mean velocity, peak velocity, time to peak velocity and smoothness [26].

Movement time was defined as the time taken to complete one movement segment. Mean velocity was calculated as the mean of the velocities recorded during the whole movement segment. The maximum absolute velocity recorded during each movement segment was taken as peak velocity. The time to peak velocity was expressed in percentage of movement time, and reflects the movement strategy. This measure distinguishes between the relative time spent during the first visually triggered outward movement until the peak velocity is reached (feedforward), and the second half of the movement toward the target that requires more precision in order to touch the target (feedback). In the second half of the movement, subjects tend to make multiple attempts to reach the target, resulting in the formation of spider-web like patterns in the movement trajectory [26]. The smoothness metric was calculated by counting the number of velocity peaks in a movement segment. The velocity profile was first searched for local minima and maxima. When the difference between a minimum and the next maximum exceeded the cut-off limit of 20 mm/s, it was counted as a velocity peak. Additionally, the time between two subsequent peaks had to be at least 150 ms. Each kinematic variable was calculated as means of all 31 movement segments for the entire task.

### Clinical assessments

The sensorimotor impairment was assessed using FMA-UE, which comprises four subscales (arm, wrist, hand, coordination) in the motor domain [7, 27]. The motor domain is designed to assess volitional movements within and without synergies in post-stroke upper limb. A maximum score of 66 in FMA-UE indicates normal arm function. FMA-UE has excellent reliability [28–30] and high degree of construct validity, particularly with Action Research Arm Test (ARAT) [31].

The activity capacity was assessed using ARAT [7]. ARAT includes 19 items divided into four hierarchical subsets (grasp, grip, pinch, gross movement). The scoring is based on the ability needed and time required for grasping and moving different object to different locations. A maximum score of 57 in ARAT indicates normal performance. ARAT has excellent reliability [32–34].

NIHSS scoring [35] was used to determine the severity of stroke impairment at the time of hospital admission. Modified Ashworth Scale (MAS) was performed to assess muscle spasticity [36]. Demographic data (age, gender, dominant hand), relevant clinical characteristics (time, location and nature of stroke) and the non-motor domains of FMA-UE (sensation, passive joint motion and pain) were also recorded.

### Statistical data analysis

The statistical data analyses were done using IBM Statistical Package for Social Sciences™ (SPSS) version 24 for Windows. Descriptive statistics, such as means, standard deviations and ranges were calculated for the study population. The amount of variance in FMA-UE and ARAT that can be explained by the kinematic variables were determined by multiple regression with forward addition. All five kinematic variables were considered for analysis along with confounding variables of age, side of the paretic arm, time since stroke and severity of impairment (NIHSS score) at stroke onset. Box-plots were plotted for all variables and no outliers greater than 3 interquartile range were found in any of the variables, thus ensuring that the regression models shall be less sensitive to outliers. The distributions of all variables were close to normal. Pearson’s correlation coefficient was used to determine the correlation between pairs of all independent variables, and those with correlation coefficient greater than 0.7 were not included in the same model [37].

Univariate regression was done at first, from which variables with *p*-values less than 0.2 were added one at a time in multiple regression model building, starting from the variable with the lowest *p*-value. These variables were retained in the model if the *p*-values were below 0.2. However, in the final models, only variables with a *p* < 0.05 were retained. Each of the confounding variables were added, one at a time, to the final model and were retained when they made significant contribution to the model, verified by an increase of R^2^ by at least 5% (*p* < 0.05). The adjusted explained variances (R^2^) with *p*-values, unstandardized regression coefficients (B) and *p*-values of each variable were reported. The model assumptions were verified by means of residual analysis, variance inflation factor (VIF) and predicted probability plots. A plot of standardized residuals against predicted values showed random pattern, VIF was less than 2 and predicted probability plots of standardized residuals showed close to normal distribution in all regression models.

## Results

The demographics, clinical data and descriptive statistics of kinematic variables from target-to-target task are shown in Table 1. Multicollinearity was observed between mean and peak velocity (r = 0.86) as well as between movement time and number of velocity peaks (r = 0.87). Significant correlations were found with FMA-UE and movement time (0.40), mean velocity (0.37), number of velocity peaks (− 0.35) and peak velocity (0.28). ARAT showed significant correlation with movement time (− 0.40), number of velocity peaks (0.36) and mean velocity (0.28).

**Table 1:** Demographic data and clinical characteristics of individuals with stroke at the time of inclusion

Demographic data and clinical characteristics	Mean ± SD, *n* (%) or median (IQR) (*n* = 64)
Age	65.7 ± 13.4
Female	26 (41%)
Time since stroke median (in months)	0.3 (0.9)
Time since stroke:	
day 3/day 10, n	29/16
week3/week 4/week 6, n	2/6/3
month 3/month 6/month 12, n	4/2/2
Ischemic stroke	52 (81%)
Haemorrhagic stroke	12 (19%)
NIHSS score at hospital admission, *n* = 61	4 (5)
Right hand dominant	61 (95%)
Right hemiparesis	27 (42%)
Left hemiparesis	37 (58%)
Fugl-Meyer Assessment – Upper Extremity (0–66)	55 ± 9, 57 (10)
Action Research Arm Test (0–57)	47 ± 9, 50 (10)
Decreased sensation (≤ 11 points, FMA)	17 (27%)
Impaired passive joint motion (≤ 23 points, FMA)	16 (25%)
Pain during passive movements (≤ 23 points, FMA)	15 (23%)
Spasticity of elbow or wrist joint (≥1 point, MAS)	5 (8%)
Kinematic variables from the target-to-target task	
Movement time (s)	2.31 ± 0.98
Mean velocity (mm/s)	146.6 ± 55.1
Peak velocity (mm/s)	374.1 ± 132.4
Time to peak velocity (%)	31.31 ± 9.0
Number of velocity peaks	4.23 ± 1.84

Abbreviations: *SD* Standard deviation, *IQR* Interquartile range, *FMA* Fugl-Meyer Assessment of Sensorimotor Function

Results of univariate regression analysis of the kinematic variables against FMA-UE and ARAT are shown in Table 2.

**Table 2:** Univariate regression analysis of the independent variables against the two dependent variables (FMA-UE and ARAT)

	Upper limb impairment (FMA-UE)			Upper limb activity capacity (ARAT)		
Independent variables	Unstandardized B	Adjusted R^2^	*p*-value	Unstandardized B	Adjusted R^2^	*p*-value
Movement time	−3.13	0.11	**0.008**	−3.56	0.13	**0.002**
Mean velocity	56.46	0.11	**0.007**	38.81	0.04	**0.07**
Peak velocity	15.43	0.03	**0.08**	6.43	0.008	0.47
Time to peak velocity	0.14	0.02	0.27	0.05	0.014	0.71
Number of velocity peaks	−1.51	0.08	**0.02**	−1.67	0.10	**0.007**
Age at onset	−0.06	− 0.009	0.51	− 0.08	− 0.002	0.35
Side of stroke affected arm	2.27	−0.001	0.33	−0.37	−0.02	0.88
Time since stroke (days)	−0.03	0.07	**0.04**	−0.03	0.03	**0.11**
Severity of impairment at onset (NIHSS score)	−0.37	0.03	**0.11**	−0.35	0.02	**0.13**

Abbreviations: *FMA-UE* Fugl-Meyer Assessment – Upper Extremity, *ARAT* Action Research Arm Test, *B* Regression coefficient, *R*^*2*^ Explained Variance. The *p* values of variables included in the multivariate analysis are given in bold numbers

Three final models were obtained from multiple regression, one with FMA-UE and two with ARAT as dependent variable. Mean velocity and number of velocity peaks explained 11% (F = 7.89, *p* = 0.007) and 9% (F = 6.17, *p* = 0.016) of the FMA-UE score uniquely and 16% (F = 7.18, *p* = 0.002) combined (Table 3). The movement time alone explained 13% (F = 10.20, *p* = 0.002) and the number of velocity peaks 10% of the ARAT score (F = 7.71, *p* = 0.007). The confounding variables did not influence any of the three final models.

**Table 3:** Multiple regression analysis of the kinematic variables and confounding variables against the two dependent variables (FMA-UE and ARAT)

Independent variables	Unstandardized B	Partial unique contribution	*p*-value of the variable	Adjusted R^2^	Model *p*-value
FMA-UE as dependent variable					
Mean velocity	53.26	0.11	0.008	0.16	0.002
Number of velocity peaks	−1.40	0.09	0.019		
ARAT as dependent variable (Model 1)					
Movement time	−3.56	–	–	0.13	0.002
ARAT as dependent variable (Model 2)					
Number of velocity peaks	−1.67	–	–	0.10	0.007

Abbreviations: *FMA-UE* Fugl-Meyer Assessment – Upper Extremity, *ARAT* Action Research Arm Test, *B* Regression coefficient, *R*^*2*^ Explained Variance

## Discussion

This study examined the association between kinematic variables obtained from target-to-target pointing task and upper extremity impairment and activity capacity limitation assessed with clinical scales in individuals with mild to moderate impairment after stroke. The results showed that mean velocity and number of velocity peaks were associated with upper extremity impairment, and they together explained 16% of FMA-UE score. Movement time and number of velocity peaks were associated with activity capacity, explaining 13 and 10% of ARAT score respectively. Age, side of the paretic arm, time since stroke and severity of stroke impairment (NIHSS score) at stroke onset did not influence these associations in a significant way.

The results from this study showed that kinematic variables could only explain a part of variance in clinical scores, although their associations were statistically significant. Kinematic variables explained slightly higher variance of FMA-UE score (16%) than ARAT score (10%). This is in line with the hypothesis that kinematic measures would show stronger correlation with impairment than activity capacity assessment, although the difference is small and further studies are needed to verify this. There are no previous data available on correlations between clinical scores and end-point kinematics obtained from haptic device during a pointing task in individuals with stroke. However, some data has been published in kinematic studies which use motion capture systems and robotic devices. Both the tasks used and the results from these studies vary considerably, and they show that kinematic variables explain 13 to 57% of the clinical scores.

A previous study showed that the smoothness measure derived from motion capture data in a reach-to-grasp task was the only significant kinematic variable among others for explaining FMA-UE score, but the exact partial correlations were not reported [14]. Similarly, smoothness and movement time explained 14 and 13% of variance in FMA-UE and 52 and 57% in ARAT in a drinking task in stroke [16]. In addition to the difference in measurement systems, these studies also differed from the current by task constraints. In addition, the task in these motion capture studies was performed in comfortable speed and had low constraint on time and accuracy as opposed to the task in the current study. For quick pointing task using motion capture, movement time explained 14% of the variance in FMA-UE [18] and 36% of the Wolf Motor Function Test –Functional Ability Score (WFMT-FAS), which is another activity capacity test [15, 38].

Robotic studies have also investigated associations between clinical scores and kinematic variables [12]. The tasks used in robotic studies often include pointing or reaching from a target to another, which is more similar to the task used in the current study. However, the movements executed in the robot-assisted devices are commonly planar 2D weight-supported movements, which differs from free 3D space movements. In a study using MIT Manus robot, a set of kinematic and kinetic variables extracted from unconstrained reaching, circle drawing and push-down tasks together explained 40% of variance in FMA-UE score [11]. The higher explanatory value for this study is probably because of the larger number of tasks analyzed and higher number of variables extracted, including kinetic variables. Moderate correlation (r = − 0.50) was reported between the WFMT-FAS and number of velocity peaks in a study where pointing task was evaluated using an end-effector robotic device [10]. A recent review examined correlations between kinematic variables from robot-assisted devices and FMA-UE [12]. The correlation coefficients for mean velocity varied between 0.01 and 0.7 and number of velocity peaks between 0.02–0.5 in different tasks.

Taken together with the findings from previous studies with similar set-up and current research, it can be concluded that only a part of variance in FMA-UE and ARAT is explained by end-point kinematics. There might be several reasons for these findings. A common understanding is that traditional clinical scales, such as FMA-UE and ARAT lack sensitivity for detecting smaller differences in movement quality and performance [39, 40]. This thought is supported by previous research in stroke populations showing that end-point kinematics are effective in discriminating between groups with different impairment levels [9, 10, 26, 41, 42] and in detecting change and improvement over time [43–45]. The data from current and past studies also suggests that association between endpoint kinematics (smoothness and movement time) and activity capacity, as measured with ARAT, might be stronger in natural reach-to-grasp tasks than in fast and accurate pointing tasks [15, 16]. One possible explanation for this might be that movement performance in self-paced reach-to-grasp tasks is more similar to what is assessed in ARAT compared to the fast and accurate pointing task. Therefore, future studies using kinematic analysis of pointing task with speed and accuracy constraints should also include clinical scales with similar task constraints when investigating correlations between the measures. The findings of the current study, in concurrence with previous studies showed that smoothness, movement time and mean velocity were the end-point kinematic variables that showed strongest associations with post stroke upper limb impairment and activity capacity, as measured with FMA-UE and ARAT. This suggests that these variables could be recommended when selecting measures for quantification of movement performance in fast target-to-target tasks in people with stroke.

The low variance explained by kinematic variables in the current and other similar studies could also be related to the relatively small workspace used in haptic devices compared to the range of movement required for completing the shoulder items of the FMA-UE. Another limitation is that the compensatory trunk movement can’t be differentiated from the whole arm movement when using an end-effector device. Interestingly, there is also some evidence that movement performance measures, such as movement time during Nine Hole Peg Test, might differ when the task is done in a virtual or in a real environment [46]. Additionally, it is possible that the selected variables capture kinematic information incompletely. Hence, more of non-redundant kinematic variables may have to be identified for the exhaustive characterization of all aspects of upper limb movements in stroke [11].

When a task needs to be performed in both fast and accurate manner, there is a tradeoff between speed and accuracy [47]. Due to this tradeoff, individuals with stroke may have been unable to reach the target in their first attempt and may have had to apply corrective actions, aided by feedback control [48]. The spider-web patterns seen in the movement trajectory of the haptic-enabled target-to-target pointing task along with varying time to peak velocity, verifies this phenomenon [26]. There is evidence that movements made towards smaller targets like used in the present study rely more on feedback control while movement towards larger objects rely more on feedforward control [49]. Thus, the movement strategy used in the target-to-target task differs from strategies used in most of the tasks performed within the FMA-UE and ARAT. Therefore, future research, in addition to scales measuring general motor function and activity capacity of the upper limb, should also include clinical scales that match the task constraints better. When rehabilitation after stroke is assessed using traditional clinical scales alone, it is likely that the focus of therapy and outcome of stroke research trials falls predominantly on improving the scores as measured by these scales. In order to obtain a comprehensive representation of all aspects of upper limb functioning, it is important to integrate kinematic assessment with clinical scales for assessment of stroke.

The strength of this study is the relatively large sample of individuals with acute stroke, where 67% of the participants were included within first 10 days of stroke onset. Such an unselected group with a large number of persons with acute stroke has rarely been reported in previous studies that examine concurrent validity of kinematic variables. The haptic device used in the current study, with its 6 degrees of freedom, captured kinematics in a 3D space, while most other studies use end effectors that are restricted to planar movements. The target-to-target task was reported as entertaining and motivating [50], indicating that it was well-accepted by the participants. The force feedback provided by the haptic device in addition to visual feedback from VR, helped to mimic the real-world scenario of pushing a real solid object, such as a button. The participants needed to have some functional ability in order to perform the target-to-target task, due to which only those with mild to moderate stroke impairment (32–65 FMA-UE score) could be included in this study.

## Conclusions

Kinematic variables of movement time, velocity and smoothness from a target-to-target pointing task are associated with upper limb impairment and activity capacity. These variables can, however, explain only a part of the variance captured by using clinical observational scales of FMA-UE and ARAT. This finding reinforces the importance of multi-level assessment using both kinematic analysis and clinical scales in upper limb evaluation after stroke.

## Data Availability

The dataset used and/or analyzed during the current study is not publicly available because further data analysis is ongoing. But, the dataset is available from the principal investigator, Katharina S Sunnerhagen **(**ks.sunnerhagen@neuro.gu.se) on reasonable request.

## Abbreviations

3D: Three dimensional
ARAT: Action Research Arm Test
B: Unstandardized Regression Coefficient
FMA-UE: Fugl-Meyer Assessment of Upper Extremity
ICF: International Classification of Functioning, Disability and Health
NIHSS: National Institute of Health Stroke Scale
r: Correlation Coefficient
R^2^: Adjusted Explained Variance
SALGOT: Stroke Arm Longitudinal Study at the University of Gothenburg
SPSS: IBM Statistical Package for Social Sciences
VR: Virtual Reality
WMFT-FAS: Wolf Motor Function Test – Functional Ability Score
